# Pointed‐snout wrasse builds nest on *Sarcotragus foetidus* sponges

**DOI:** 10.1002/ecy.4531

**Published:** 2025-01-22

**Authors:** Francesca Strano, Francesco Tiralongo, Valerio Micaroni

**Affiliations:** ^1^ Department of Biological and Environmental Sciences and Technologies University of Salento Lecce Italy; ^2^ School of Biological Sciences Victoria University of Wellington Wellington New Zealand; ^3^ Ente Fauna Marina Mediterranea, Scientific Organization for Research and Conservation of Marine Biodiversity Avola Italy; ^4^ Department of Biological, Geological, and Environmental Sciences University of Catania Catania Italy; ^5^ National Research Council, Institute of Marine Biological Resources and Biotechnologies Ancona Italy

**Keywords:** copy‐cat behavior, nesting behavior, sponge, wrasse

Fishes belonging to the genus *Symphodus*, commonly called wrasses (Labridae family), exhibit diverse nesting behaviors among different species, ranging from elaborate to rudimentary nests, and in some cases no nests at all (Hanel et al., 2002; Warner & Lejeune, 1985). The nests, when built, are primarily constructed using algae of various species, which provide a secure environment for egg deposition and protection, playing a crucial role in the reproductive success of these species (Quignard & Pras, 1986). The Pointed‐snout wrasse (*Symphodus rostratus*), a common Mediterranean species, exhibits complex nesting behaviors (Hanel et al., 2002), which are essential for mate selection (Alonzo & Heckman, 2010).

During a rebreather training dive along the Apulian coast (Santa Caterina, Ionian Sea; 40°08′15.4″ N, 17°58′52.3″ E), the 20th of March 2024, at a depth between 12 and 17 m, we encountered large specimens (40 cm in diameter) of the sponge *Sarcotragus foetidus* with systematically arranged mounds of biogenic material, including shell, sea urchin test, and bryozoan fragments on their surface (Figure 1). In this context, we observed a mature male of the Pointed‐snout wrasse actively transporting these fragments and placing them on one of these sponges (Video S1), engaging in nest‐building behavior (Taborsky et al., 1987). Throughout our dive, we documented nine instances of *S. rostratus* nests constructed on *S. foetidus*, each between 30 and 40 cm in diameter. In one case, we observed and filmed (GoPro HERO 8 Black) a female engaging in mating behavior with a reproductive male (Video S2). This observation is significant, as the use of calcareous biogenic materials and the selection of sponges as a nesting substrate in *S. rostratus* had never been reported until now. The Pointed‐snout wrasse indeed, typically builds its nets using algae as the primary material, selecting areas rich in vegetation. This species is commonly observed constructing nests among algal beds, which provide both camouflage and structural support to eggs. The finding of several nests on different sponges within the same site suggests the possibility of observational learning or “copy‐cat” behavior among reproductive male *S. rostratus*. Copying behavior have been documented in Ocellated wrasse (*Symphodus ocellatus*), where female mate choice copying can influence sexual selection (Alonzo, 2008), and if confirmed, this would represent a novel example of observational learning in the nesting behaviors of *S. rostratus*.

**FIGURE 1:**
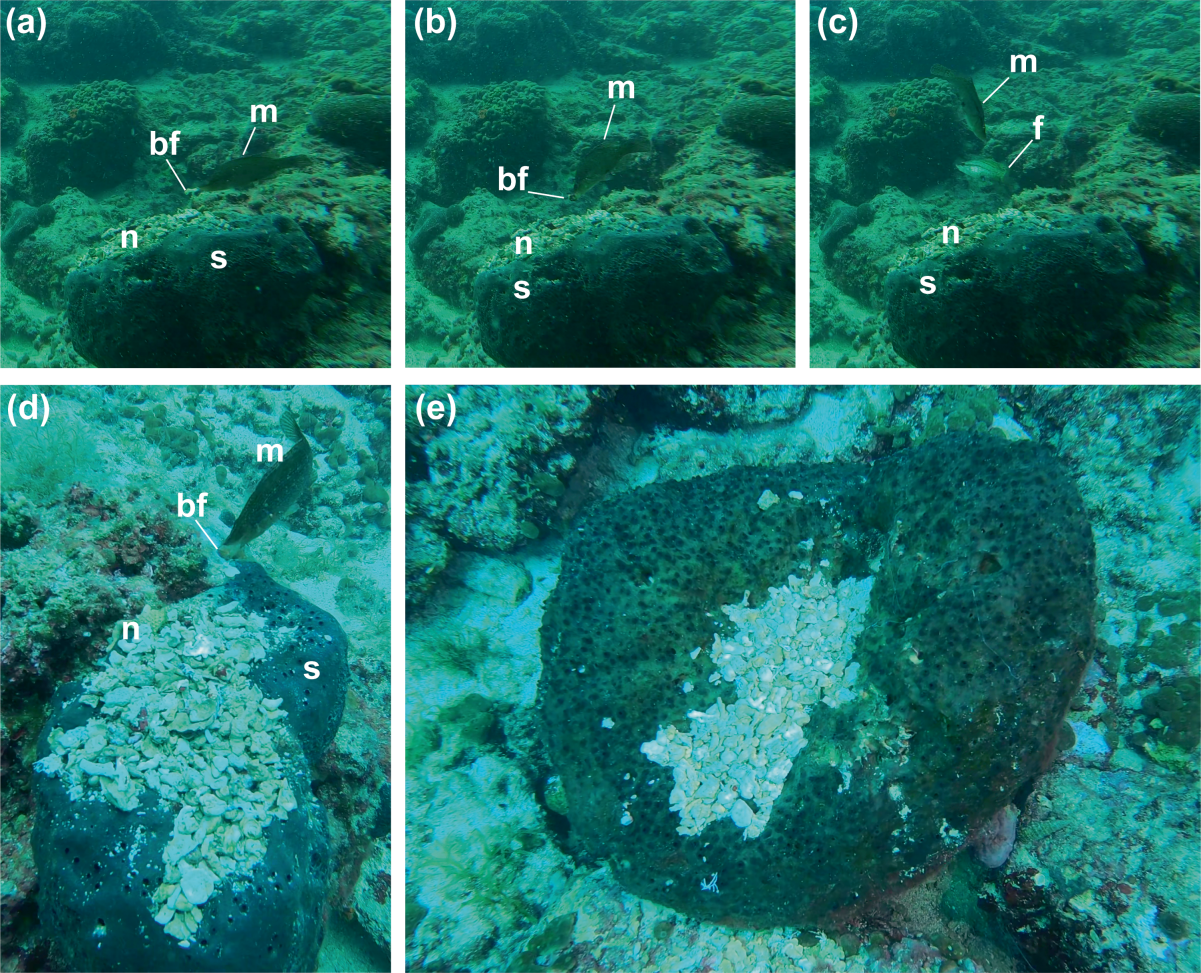
Pictures depicting males of *Symphodus rostratus* nesting on specimens of *Sarcotragus foetidus* (20 March 2024; Santa Caterina, Ionian Sea; 40°08′15.4″ N 17°58′52.3″ E): (m) male; (bf) biogenic fragment; (f) female; (s) sponge; (n) nest. Photos by Francesca Strano and Valerio Micaroni.

Sponges play crucial ecological functions in marine ecosystems, often providing habitats for other organisms at various spatial scales (Bell et al., 2023). Deep‐sea glass sponge reefs are known to be refuge and nursery grounds for demersal fish (Barthel, 1997; Maldonado et al., 2017). For example, the Antarctic painted notie (*Nototheniops larseni*) has been reported to lay egg masses in the central cavity of the glass sponge *Rossella nuda* (Konecki & Targett, 1989). Other examples of fish using sponges as nesting sites include the Sea raven (*Hemitripterus americani*), which lays egg masses between the branches of the sponge *Chalina* sp. (Warfel & Merriman, 1944), and the Bigmouth sculpin (*Hemitripterus bolini*) using several sponge species as nests in the Gulf of Alaska and Bering Sea (Busby et al., 2012). In addition, two fish species from the Japanese coasts, the Silver‐spotted sculpin (*Blepsias cirrhosus*) and the Filefish (*Brachaluteres ulvarum*), also lay eggs within the sponge tissue and in the sponge suboscular cavity, respectively (Akagawa et al., 1995; Munehara, 1991). However, the use of sponges as direct nesting sites for wrasses is scarcely reported in the literature.

This unexpected nesting behavior raises several questions. Could the water circulation from the sponge's pumping activity enhance eggs ventilation? Sponges can pump substantial volumes of water daily, and this water movement could provide excellent ventilation for fish eggs during development. Does the typical central depression of *S. foetidus* (Pulido Mantas et al., 2024) provide an advantageous structure for nest building? The distinctive central depression occurring in this sponge may help the fish retain biogenic fragments forming the nest and keep eggs in place, protecting them from wave action. Could this behavior be an adaptive response to environmental changes, such as alterations in algal availability or shifts in breeding season timing due to climate change or other environmental alterations? Climate change often causes mismatches in ecological interactions, so it is possible that this fish is using biogenic materials instead of algal fragments due to the absence of those algae, which could also be attributed to environmental changes.

Our observation corroborates the occurrence of complex behaviors in wrasses and indicates a potential new ecological interaction between sponges and fish reproduction in coastal environments. We only recognized the unusual behavior's importance months after documenting it, and this prevented our return to the site. Indeed, additional research is necessary to determine the frequency and distribution of this behavior, its potential benefits, and its implications for both *S. rostratus* reproduction and *S. foetidus* ecology. This observation presents opportunities for investigating the adaptability of fish breeding strategies and the diverse ecological functions of sponges in marine ecosystems.

## CONFLICT OF INTEREST STATEMENT

The authors declare no conflicts of interest.

## Supporting information


Video S1.

Video S2.

Video S1 metadata.

Video S2 metadata.

## Data Availability

Video files are also provided in Strano (2024) in Figshare at https://doi.org/10.6084/m9.figshare.27914004.
