# Supplementary material for: Pointed‐snout wrasse builds nest on Sarcotragus foetidus sponges
Source: Ecology. 2025 Jan 22;106(1):e4531. doi: 10.1002/ecy.4531 (PMC11751700; doi:10.1002/ecy.4531)
Supplement: Supplementary file 4 — Video S2 metadata. [file ECY-106-e4531-s001.pdf]

Journal: Ecology

Pointed-snout wrasse builds nest on *Sarcotragus foetidus* sponges

Francesca Strano, Francesco Tiralongo, Valerio Micaroni

Video S2 Metadata. Mature male of Pointed-snout wrasse (*Symphodus rostratus*) actively transporting biogenic fragments and placing them on *Sarcotragus foetidus* sponge and engaging in mating behavior with a reproductive female. Filmed by Francesca Strano and Valerio Micaroni.
